# Pre- and post-operative Wisconsin card sorting test performance in patients with temporal lobe epilepsy due to hippocampal sclerosis

**DOI:** 10.1590/s1980-57642008dn10200010

**Published:** 2007

**Authors:** Luciana Tisser, Andre Palmini, Eliseu Paglioli, Mirna Portuguez, Ney Azambuja, Jaderson Costa da Costa, Eduardo Paglioli, Carolina Torres, Jose Victor Martinez

**Affiliations:** 1 MSc, Porto Alegre Epilepsy Surgery Program, Services of Neurology and Neurosurgery, Hospital São Lucas da Pontificia Universidade Católica do Rio Grande do Sul (PUCRS).; 2 MD, PhD, Division of Neurology, Department of Internal Medicine and; 3 Division of Neurosurgery, Department of Surgery, Faculty of Medicine, PUCRS, Porto Alegre, Brazil.

**Keywords:** temporal lobe epilepsy, hippocampal sclerosis, executive dysfunction, Wisconsin card sorting test, epilepsy surgery

## Abstract

Patients with temporal lobe epilepsy due to hippocampal sclerosis (TLE/HS) have a distinct neuropsychological profile, but there is still debate on whether executive dysfunction is part of this profile and also whether temporal lobe surgery can modify this dysfunction.

**Objective:**

To study the presence and reversibility of executive dysfunction in patients with unilateral TLE/HS.

**Methods:**

Twenty-five patients with refractory seizures due to TLE/HS underwent presurgical evaluation which included the application of the Wiconsin Card Sorting Test (WCST). Nineteen were re-evaluated in follow up, at least 6 months after selective amygdalo-hippocampectomy (SAH). Twenty-two control subjects matched for age and education also performed the WCST.

**Results:**

Sixteen of the 25 patients (64%) completed fewer than four categories in the WCST whereas only 4 of the 22 controls (18%) did not complete at least four categories (p<0.005). In addition, the performance of the patients involved significantly more perseverative responses and errors compared to controls. The patient group demonstrated significant post-operative improvement in many measures of the WCST following SAH.

**Conclusions:**

These findings support the presence of executive dysfunction in patients with TLE/HS and suggest that such dysfunction can be partially reversed by selective resection of epileptogenic mesial temporal structures.

## Abstract (pt)

Pacientes com epilepsia de lobo temporal associada à esclerose hipocampal (ELT/EH) têm um perfil neuropsicológico em sua maior parte bem reconhecido, embora discuta-se se manifestações de disfunção executiva acompanham este perfil e se esta disfunção poderia ser modificada por cirurgia da epilepsia de lobo temporal.

**Objetivo:**

Estudar a presença e reversibilidade da disfunção executiva em pacientes com ELT/EH unilateral.

**Métodos:**

Vinte e cinco pacientes com crises refratárias associadas à ELT/EH foram submetidos à avaliação pré-cirúrgica que incluiu a aplicação do Teste de Seleção de Cartas de Wisconsin (TSCW). Dezenove foram reavaliados um mínimo de 6 meses após amígdalo-hipocampectomia seletiva (AHS). O TSCW foi também aplicado a 22 indivíduos controles pareados por idade e nível educacional.

**Resultados:**

Dezesseis dos 25 pacientes (64%) completaram menos de quatro categorias no TSCW, enquanto que apenas 4 dos 22 indivíduos do grupo controle (18%) tiveram este mesmo nível e disfunção (p<0.005). Além disto, a performance dos pacientes aresentava significativamente mais respostas e erros perseverativos do que os controles. Analisados como um grupo, os pacientes apresentaram melhoras significativas em várias medidas do TSCW após AHS.

**Conclusão:**

Estes achados apontam para a presença de disfunção executiva em pacientes com ELT/EH e sugerem que esta disfunção pode ser parcialmente revertida pela ressecção seletiva das estruturas temporais mesiais epileptogênicos.

Patients with temporal lobe epilepsy (TLE) often present refractory seizures and are thus referred for presurgical evaluation.^1,2^ In a significant proportion of such cases, magnetic resonance imaging (MRI) shows unilateral hippocampal atrophy and loss of internal architecture, manifested as an abnormal signal of the hippocampus in T1, T2, and Fluid-attenuation inversion recovery (FLAIR) images. These findings are collectively indicative of hippocampal sclerosis (HS), the most common etiology of TLE in adolescents and adults.^1,4-6^

Patients with TLE/HS tend to have a low quality of life (QoL), apparent both at clinical interviews and on more formal QoL questionnaires.^8-10^ This negative impact stems mostly from the refractory seizures, but is also compounded by high rates of depression and anxiety, unemployment, memory difficulties, and an overall reduced ability to deal with life stressors.^11^ An issue that is largely unexplored is the possibility that *executive dysfunction* may affect QoL in patients with TLE/HS. This relative negligence is probably due to the location of the lesion and of the epileptogenic zone in the temporal lobe, theoretically sparing the frontal lobe networks reputedly involved with executive functions. Therefore, a massive amount of data has accumulated concerning memory function and dysfunction in TLE,^12-14^ whereas the data on executive functioning is limited. Nonetheless, anatomical connectivity between temporal and frontal lobe structures provides a tentative link between TLE and executive functions,^15^ which has began to be explored. A few studies in the last decade^16-19^ have shown that patients with TLE of several different etiologies may have abnormal WCST scores.

When patients with TLE/HS have refractory seizures, epilepsy surgery is a highly effective treatment, both in terms of seizure control and improvement in QoL measures, translating into better overall functioning.^1,2,6^ The latter is believed to be directly dependent upon and related to seizure control; however, the possibility that an improvement in cognitive, including executive, functioning may also play a role has not been formally studied.

In recent years, we have begun to explore these issues at the Porto Alegre Epilepsy Surgery Program. In a first step, we followed a large cohort of patients for more than 10 years and reported that surgery for TLE/HS is associated with stable levels of seizure freedom in around 85-90% of patients.^6,7^ In a second set of studies, we then prospectively addressed the presence, associated features, and reversibility of executive dysfunction evaluated with a comprehensive test battery in a group of patients with TLE/HS (Tisser et al., in preparation) undergoing presurgical evaluation and epilepsy surgery. As part of these latter studies, the current work reports on the results of the single most informative test of executive function in patients with epilepsy, namely the Wisconsin Card sorting Test (WCST), and focus on the pre- and post-operative performance of this cohort with TLE/HS. These studies will hopefully pave the way for a future project on the role of executive functions in QoL measures of patients with TLE/HS, pre and post surgery.

## Methods

We studied 25 patients with TLE, refractory seizures, and MRI features characteristic of unilateral HS, who were consecutively evaluated on the Porto Alegre Epilepsy Surgery Program, Hospital São Lucas da Pontifícia Universidade Católica do Rio Grande do Sul (PUCRS) in Porto Alegre, from October 2005 to May 2006. All patients underwent a comprehensive presurgical evaluation to localize the epileptogenic zone, determine neuropsychological status, and rule out major psychiatric disorders through structured interviews.^20^ All were able to fully cooperate in the neuropsychological test battery and all other procedures. Two other patients with TLE/HS evaluated in the same period were excluded because they had an estimated IQ of <79. The clinical, neurophysiologic and neuroimaging findings leading to the diagnosis of TLE/HS have been previously published.^6,7^ Briefly, all patients had (i) clinical features characteristic of mesial TLE;^21-23^ (ii) interictal scalp/sphenoidal EEGs with unilateral or bilateral independent anteromesial temporal epileptiform discharges; (iii) at least one electroclinical seizure on scalp/sphenoidal video-EEG monitoring with clearcut unilateral temporal onset; and (iv) 1.5 Tesla MRI with at least two of the following: hippocampal atrophy, decreased intrahippocampal signal on T1-weighted images, or increased intrahippocampal signal on T2-weighted and fluid-attenuated inversion recovery (FLAIR) images. Age at evaluation ranged from 28 to 48 years (mean, 36.2), mean epilepsy duration was 25.7 years, and 13 were women. The lateralization of the epileptogenic zone, the degree of predominance between the temporal lobes in the distribution of the interictal spikes in patients with bitemporal independent discharges, and the antiepileptic drugs being used were also noted.

### Controls

Twenty-two healthy individuals, without any history of craniocerebral trauma, seizures or previous neurologic or psychiatric disorders were randomly selected as controls from the technical staff of another Hospital in Porto Alegre. They were matched for age and educational status with the epileptic patients. Seventeen were women and the age range of the control group spanned from 20 to 48 years (mean, 32.8, SD, 8.7).

### Neuropsychologic evaluation

Intelligence quotient (IQ) was estimated through the digits, vocabulary, and cubes subtests of the Wechsler Adult Intelligence Scale - III^rd^ Edition - Revised (WAIS-III-R), Portuguese version (24), while the Wechsler Memory Scale - Revised (WMS-R) was used to evaluate episodic memory.^25^ Results of the latter and their relation with WCST performance are to be presented elsewhere (Tiser et al, in preparation). Executive functions were evaluated through the WCST, Portuguese version,^26^ and although this test is still in the process of validation for the Brazilian adult population, its extensive clinical application allows an inference of clinically-relevant criteria. For instance, a total number of completed categories in the WCST of ‘less than four’ is universally accepted as indicative of executive dysfunction for the age range of the patients and controls studied in the current investigation. The presence and quantification of symptoms of depression and anxiety were determined through the Beck Inventary of Depression and Anxiety, Portuguese version.^27^

### The WCST

This is a time-honored test to evaluate executive functions, and test procedures have been published elsewhere.^28^ Briefly, the subject has to formulate a strategy to combine each of 128 cards consecutively drawn from a deck with one of four index cards which vary according to number, geometrical design, and color. The only feedback the subject receives from the examiner is whether his/her choice was correct or incorrect and he/she has to use this information to combine ‘each next card’. Specifically, no feedback is given on why the choice was considered *correct* or *incorrect* by the examiner. A *category* is deemed *complete* when the subject succeeds in *correctly combining 10 consecutive cards* drawn from the deck. Following this, and unbeknownst to the subject, the ‘correct’ strategy of pairing is changed (for instance, from *color* to *geometrical form*) and this has to be realized by the subject who then has to pair the cards according to this new strategy. The test evaluates abilities such as mental flexibility and impulse control. In particular, it probes the tendency of the subject to persevere in a strategy no longer valid despite receiving repeated ‘incorrect’ feedback and the ability to retain the newly valid strategy ‘on-line’ at the time of each choice.

We evaluated and analyzed the following WCST variables:

(i) number of correctly completed categories, to a maximum of 12;
(ii) number of ‘correct’ pairings;
(iii) number of ‘incorrect’ pairings;
(iv) number of perseverative pairings, i.e., pairings in which the subject perseveres in *any random* strategy, despite repeated feedback that the choice was ‘incorrect’; and
(v) number of perseverative ‘errors’, i.e., incorrect pairings in which the subject perseveres in choosing according to the *immediately previous strategy* which evoked correct feedback.

### Post-operative evaluation

Post-operative evaluations of the WCST were performed between 6 and 12 months after surgery in 19 of the 25 patients. The other 6 could not be included because the interval between the operation and re-testing was shorter than 6 months.

### Surgical procedure

All patients underwent a selective amygdalo-hippocampectomy (SAH) in which unilateral mesial temporal structures were removed according to the technique originally described by Niemeyer.^29^ The temporal horn of the lateral ventricle was reached through a 2.0 to 2.5 cm incision in the second temporal gyrus, and excision of the amygdala, hippocampus and parahippocampal gyrus proceeded with aspiration of the amygdala, the anterior 2-3 cm of the hippocampus and of the parahippocampal gyrus extending posteriorly to the mid-mesencephalic level.^7^ The left mesial temporal structures were resected in 15 patients.

### Framework of the study and statistical analysis

We first compared the scores of the patients with those of the controls on the depression and anxiety scales and, more specifically, on the WCST. In a second step, we focused on the patients and compared WCST performances before and after epilepsy surgery. All subjects consented to participate in the study, which was approved by the Ethics committee of the Hospital São Lucas da PUCRS.

Quantitative variables were described through means, standard deviations of the mean and Pearson’s correlation coefficient. The Student’s t test was used to for comparison between means, the Kolmogorov-Smirnov for testing the normal distribution of quantitative variables, and the Mann-Whitney to compare the distribution of non-parametric variables. The SPSS statistical package, version 13.0 was used for all statistical analyses and a level of 0.05 was considered to be statistically significant.

## Results

Patients’ mean age at evaluation was 36 and for controls 32 years, where less than one third in both groups had completed secondary school. Likewise, sex distribution, estimated IQ, and scores in depression and anxiety scales did not significantly differ between patients and controls (Table 1). Mean age at seizure onset and mean duration of epilepsy before operation were, 10 and 26 years, respectively. In 10 patients, a history of previous insult to the central nervous system was reported, where this took the form of febrile convulsions in 8. In 15 patients (60%) the HS was in the left temporal lobe and interictal epileptiform discharges always predominated in the temporal lobe ipsilateral to the HS. Discharges were strictly unilateral or had a large (>90%) unilateral predominance in 75% of patients. The remaining quarter had between 70 and 90% of epileptic discharges lateralized to the side of the HS. Most patients were receiving carbamazepine or phenytoin at the time of presurgical evaluation. Antiepileptic drugs and dosages were not modified during the first post-operative year.

**Table 1:** Demographic, IQ, and psychiatric fi ndings.

Variables	TLE/HS n=25	Controls n=22	p
**Age, (DP)**	36.2 (4.9)	32.8 (8.7)	0.100
**Sex, n (%)**			0.072
Male	12 (48.0%)	5 (22.7%)	
Female	13 (52.0%)	17 (77.3%)	
**Schooling, n (%)**			0.388
Incomplete elementary schooling	15 (60.0%)	10 (45.5%)	
Complete elementary schooling	3 (12.0%)	6 (27.3%)	
Complete secondary schooling	7 (28.0%)	6 (27.3%)	
**Estimated IQ, n (%)**			0.174
Inferior average	4 (16.0%)	0 (0.0%)	
Average	16 (64.0%)	17 (77.3%)	
Superior average	5 (20.0%)	4 (18.2%)	
Superior	0 (0.0%)	1 (4.5%)	
**BDI**			0.956
Minimum, n (%)	18 (72.0%)	16 (72.7%)	
Mild/moderate, n (%)	7 (28.0%)	6 (27.3%)	
**BAI**			0.123
Minimum, n (%)	23 (92.0%)	16 (72.7%)	
Mild/moderate,. n (%)	2 (8.0%)	6 (27.3%)	

n, absolute number, BDI, Beck depression inventory; BAI, anxiety inventory; TLE/HS, temporal lobe epilepsy due to hippocampal sclerosis.

### Pre-operative neuropsychological scores

Sixteen of the 25 patients (64%) completed less than four categories in the WCST whereas only 4 of the 22 controls (18%) did not complete at least four categories (p<0.005). Mean number of completed categories for the patients was 3.1 (SD, 2.0) whereas for the controls was 4. 7 (SD, 1.6) (p <0.005). With the exception of the number of total correct responses, in all other parameters evaluated in the WCST the controls fared significantly better than the patients (Table 2).

**Table 2:** Performance on the WCST by patients with TLE/HS, and by controls.

WCST parameter	TLE/HS mean (SD)	Controls mean (SD)	p
Number of completed categories	3.1 (2.0)	4.7 (1.6)	**0.005**
Total number of correct pairings	63.8 (14.9)	74.0 (9.3)	0.100
Total number of errors	56.4 (24.0)	37.8 (18.9)	**0.005**
Number of perseverative responses	47.4 (34.4)	20.8 (13.7)	**0.001**
Number of perseverative errors	38.2 (25.0)	11.6 (11.7)	**0.001**

In addition, there was no correlation between the scores on the BDI or the BAI and the scores on the WCST (data not shown).

### Post-operative results

Following selective amygdalo-hippocampectomy, the post-operative performance of patients with TLE/HS in several WCST parameters was significantly better than before operation (Table 3). This included total number of correct pairings (p=0.043), total number of errors (p=0.036), number of perseverative errors (0.004) and also of perseverative responses (p=0.002). Improvement in total number of completed categories had a trend toward significance.

**Table 3:** Comparison of pre- and post- operative performance of patients with TLE/HS on the WCST.

WCST parameter	Pre-op mean (SD)	Post-op mean (SD)	p
Completed categories	2.9 (2.0)	3.6 (1.9)	0.125
Correct pairings	62.7 (16.2)	69.9 (14.3)	0.043
Total number of errors	58.7 (24.4)	49.9 (21.7)	0.036
Perseverative responses	49.7 (36.0)	31.0 (17.6)	0.002
Perseverative errors	9.6 (26.0)	26.8 (14.2)	0.004

Females younger than 35 years at operation, whose epilepsy had started before age 12 and who had recurrent seizures for less than 25 years tended to score higher on the post-operative WCST than males who were older than 35 years at operation and whose seizures had recurred for more than 25 years. Laterality of the epileptogenic zone did not correlate with post-operative scores, although this finding should be viewed in the context of the limited patient sample. In contrast, those patients who continued on carbamazepine or phenytoin monotherapy at the time of re-testing had significantly less post-operative perseverative errors in comparison with those who continued on valproate, phenobarbital or clobazan, or on polytherapy regimens.

## Discussion

We have shown that patients with unilateral TLE/HS display abnormalities in tests of executive function and that such executive dysfunction can be partially reversed by selective resection of the epileptogenic mesial temporal lobe structures. When compared to a control group, TLE/HS patients had a significantly poorer performance in several parameters of the WCST. Other series including patients with TLE of several different etiologies have shown similar results, in that abnormal WCST scores were seen in 42 to 75% of patients.^16,17^

We believe our findings may prove important for several reasons. First, we are not aware of similar studies in an etiologically homogeneous population of patients with medically refractory TLE undergoing a selective surgical procedure. Other studies examining executive functions in TLE either included patients with different etiologies or performed more extensive resections of temporal lobe tissue.^18,30^ Because distinct etiologies of TLE may interfere with different temporal lobe structures and circuits, the etiological homogeneity of our patients allows more consistent considerations regarding the impact of functional and epileptiform abnormalities in the limbic system on the executive functions. Second, our findings may help to explain why patients with TLE/HS often have difficulties in conducting their lives which appear to extrapolate the negative impact of any recurrent seizures and the memory abnormalities. Admittedly, teasing out the putative disruptive effects of the executive dysfunction from those of the epilepsy and of the prevalent mood and memory abnormalities may be a difficult task. However, in view of the pivotal role of the executive functions in problem-solving and decision-making it would hardly be a surprise if executive dysfunction is eventually shown to impact the QoL of patients with TLE/HS. Third, this study adds further neuropsychological evidence to a growing body of cognitive, neurophysiological, metabolical, and brain perfusional research which consistently points to abnormalities in frontal lobe structures in patients with unilateral TLE/HS, thus suggesting that this disorder may impact the brain in a much more diffuse fashion than previously imagined.^19,31-35^ Finally, because the post-operative improvement in executive functions reported in this study contrasts with the less favorable results reported in similar patients undergoing non-selective resections, i.e., temporal lobectomy^18,30^ XX, our findings suggest that sparing temporal neocortical structures may be relevant in this respect.

Traditionally, QoL limitations in patients with TLE/HS have been related to the recurrent seizures and resulting stigmata and deprivations.^9^ However, the issue of whether the executive dysfunction found in these patients - at least in neuropsychological testing - is also a major contributor to limitations in QoL, warrants further investigation. There is some support for this notion in the recent literature. Getz and colleagues showed a direct association between QoL in patients with TLE/HS and the presence of negative symptoms, many of which^36,37^ related to executive dysfunction. Clearly, the hypothesis that executive dysfunction contributes to limitations of QoL in patients with TLE/HS needs to be explicitly tested, correlating symptoms with neuropsychological test scores.

A debated issue is whether the abnormalities in tests of executive function in patients with TLE/HS are due to the fact that the mesial temporal structures anatomically connected to the frontal lobes - and thus part of a fronto-temporal anatomofunctional network - are structurally and functionally abnormal or if such secondary frontal lobe dysfunction derives in a dynamic fashion from the temporo-frontal synaptic transmission of the electrical abnormalities represented by recurrent seizures and interictal temporal lobe spikes.

It is known that mesial temporal structures contribute heavily toward episodic memory and the emotional relevance of incoming stimuli. Coupled with a broader view of working memory as also incorporating previously memorized information which is temporarily made conscious to support specific decision-making, it can be conceived that hippocampal dysfunction could interfere in the executive functions.^38,39^ Likewise, alterations in the amygdala, often present in patients with TLE/HS, may interfere with emotional tuning thus affecting hierarchization of stimuli and other aspects of decision-making. Thus, theoretically at least, abnormalities in mesial temporal structures related to episodic memory and emotional processing of stimuli may contribute to executive dysfunction, irrespective of the presence or propagation of epileptic discharges into the frontal lobes.

However, irrespective of the debate on the role of the ‘fixed’ abnormalities of mesial temporal structures in the executive dysfunction of patients with TLE/HS, improvement in performance following unilateral resection of these very structures strongly suggests that the dynamic epileptiform abnormalities in temporo-frontal networks are more relevant to the executive dysfunction than the static histological abnormalities. Following selective resection of mesial temporal structures, patients committed significantly fewer overall and perseverative errors while augmenting their correct pairings (choices) in comparison to their preoperative performance. If the ‘fixed’ amygdalar and hippocampal dysfunction were the major determinants of this performance, improvements following resection of these structures would be unlikely. An additional way to prove this point is to correlate the preoperative seizure frequency with performance in the WCST. This may reveal interesting associations and inform which patients with TLE/HS are at a greater risk of developing executive dysfunction with seizure recurrence. These possibilities are to be explored in another study (Tisser et al., in preparation).

Moreover, the results of the present work are in line with those seen in previous neuropsychological PET, SPECT and invasive electrode studies in patients with TLE/HS, all suggesting a relationship between temporal epileptic discharges and seizures and electrical, metabolical, and perfusional disturbances in the frontal lobes.^19-31-35^ Studying patients with TLE/HS and controls with ictal SPECT, Van Paesschen et al.^33^ showed significant *ictal hypoperfusion* in the frontal lobes. The same group furthered these findings by adding interictal FDG-PET analysis of similar patients,^34^ showing that the most intense interictal hypometabolism was indeed in the frontal lobe ipsilateral to the mesial temporal epileptogenic zone, *and not*, as expected, in the temporal lobe itself. Furthermore, during seizures, ictal SPECT showed an extensive ring of *hypo*perfusion, maximal in the ipsilateral frontal lobe, colocalizing with the frontal areas showing maximal hypometabolism on FDG-PET. These data suggest a dynamic process including significant inhibition in the frontal lobes in patients with TLE/HS. In another recent study, Takaya et al.^35^ studied cognitive functions and interictal metabolism in 21 patients with mesial TLE. They showed that the 11 with more frequent seizures had more cognitive deficits than the 10 in whom seizures were infrequent, and that this was associated with prefrontal hypometabolism.

From a neuropsychological perspective, the pattern of abnormalities in tests of executive function suggests that patients with TLE/HS have difficulties with mental flexibility and contextual adaptation, and that the post-operative improvement in these functions may have a positive clinical impact. Data on such post-operative changes in tests of executive function is still scant but was commenced by Hermann and colleagues,^19,40^ who showed that some patients with TLE and executive dysfunction could improve after operation. Much more recently, Kim et al.^41^ compared 85 patients with mesial and 34 with lateral TLE, and found that 56% of those with mesial TLE had significant deficits in performance in the WCST. No specific variable correlated with post-operative improvement, however, those with best scores preoperatively tended to deteriorate whereas those with poorer preoperative scores had some improvement. These results support the data and the hypothesis expressed in the present study, confirming that many patients with TLE/HS have executive dysfunction and that the latter may be ameliorated with resection of the mesial temporal structures. In addition, their finding that patients with mesial and lateral neocortical TLE did not differ in their performance in the WCST suggests that temporal epileptogenicity is likely to take precedence over the mere dyfunction of mesial temporal structures in provoking frontal lobe (executive) dysfunction. On the other hand, Martin et al.^30^ did not find significant modifications in post- versus pre-operative performance in a group of 174 patients with TLE undergoing (nonselective) anterior temporal lobectomy. These negative findings were not modified either by the side of resection or the degree of seizure control. In another study on 72 patients with TLE undergoing the nonselective procedure, the same authors^18^ confirmed their negative results in terms of changes in post-operative WCST performance, even when HS was bilateral, and irrespective of the lesion type causing the epilepsies. These diverging results raise the possibility that the additional resection of the lateral temporal neocortex - which was performed in the patients who did not improve^18,30^ and yet not performed in the series in which many patients improved (41; present study) - somehow prevented a post-operative improvement in the WCST. This is not a universal finding^19,40^ and therefore this hypothesis needs to be tested prospectively.

A potential limitation of the present study is that we did not re-test the control subjects after a similar 6-month interval. This leaves open the question of whether or not a ‘learning effect’ is operative when re-applying the WCST. However, evidence exists suggesting that the results of re-testing patients with the WCST are not compromised by a learning effect. Data collected for the WCST normative manual^26^ showed the instrument to produce stable results over time, even when re-testing was performed, in as little as one month following initial exposure, providing, of course, that the rationale of the test was not disclosed to the subjects after the first application. In addition, in one of the few studies directly addressing this issue, Ingram and colleagues provide data supporting the temporal stability of most variables probed with the WCST and suggest that previous contentions suggestive of a learning effect were related to the application of the WCST to non-patient populations.^42^ Finally, we believe that if a learning curve were operant in our patients, it would be likely that performance during re-testing should have been better than we observed, despite the unequivocal improvements reported here.

As a final point, we found preliminary associations between gender, age at onset, epilepsy duration, and type of antiepileptic drug regimen and post-operative performance in the WCST. The small number of patients included in these analyses precludes bolder statements, but these preliminary findings probably warrant further study.
